# Terminologia Anatomica Compliance in Generative Artificial Intelligence Outputs: A Structured Narrative Review and Terminology-Specific Verification Checklist

**DOI:** 10.7759/cureus.116639

**Published:** 2026-09-21

**Authors:** Deepak Sharma, Anaida Singh, Ahmed Mahgoub

**Affiliations:** 1 Basic Science, Lake Erie College of Osteopathic Medicine, Erie, USA; 2 Medicine, St. George's University, St. George's, GRD; 3 Anatomy, Tanta University Hospital, Tanta, EGY

**Keywords:** anatomical terminology, anatomy education, generative artificial intelligence, medical illustration, publication ethics, terminologia anatomica

## Abstract

Generative artificial intelligence (GenAI) can produce fluent anatomical explanations and persuasive illustrations, but fluency and visual realism do not establish compliance with Terminologia Anatomica, second edition (TA2), or anatomical correctness. Unlike broader anatomy-education governance frameworks, this evidence-informed narrative review isolates terminology compliance as a distinct validation problem. It examines six connected domains: baseline human and editorial use of standardized terminology, text-based GenAI performance, image-generation fidelity, citation reliability, multilingual terminology, and publisher oversight.

Direct evidence is heterogeneous but increasingly specific. Recent studies have used dedicated terminology measures or TA2 as a reference standard for text outputs, while image studies consistently identify omissions, invented structures, incorrect relationships, and mislabeled anatomy. One anatomy-specific citation study also found substantial reference hallucination and limited PubMed identifier accuracy. These risks occur against a pre-existing background of incomplete TA adoption among anatomists and journal editors, so GenAI may reproduce or amplify inconsistencies already present in its source environment. Evidence concerning non-English TA2 output remains particularly limited.

A four-step terminology-specific checklist is therefore proposed: classify the output and its risk; verify every anatomical term against an authoritative terminology source; verify scientific content, structural fidelity, and source traceability; and document accountable human review and disclose AI assistance. Verification should be tiered by consequence: low for internal brainstorming, moderate for teaching materials, and high for summative assessment, publication, and clinical or procedural use.

The checklist is intentionally practical rather than a validated scoring instrument. Current evidence does not support treating any general-purpose GenAI system as reliably TA2-compliant without human verification.

## Introduction and background

Anatomical terminology provides a shared, internationally intelligible language for describing the human body. Terminologia Anatomica, second edition (TA2), is the current international reference produced through the Federative International Programme for Anatomical Terminology (FIPAT) of the International Federation of Associations of Anatomists (IFAA) [1,2] and is publicly accessible through the TA2 Viewer [3]. An eponym names a structure after a person rather than describing it; for example, the eponym "Fallopian tube" is commonly used for the TA2 term "uterine tube." TA2 favors standardized, descriptive terms while recording relationships among structures and limiting dependence on variable regional synonyms and eponyms.

Generative artificial intelligence (GenAI) introduces a new route by which anatomical language and imagery enter teaching, assessment, academic writing, and public communication. Large language models can generate explanations, examination questions, tables, translations, and references, while text-to-image systems can generate polished illustrations containing omitted, duplicated, displaced, or invented structures. These errors matter beyond nomenclature: inconsistent labels can mislead learners, alter assessment meaning, impair literature retrieval, and create ambiguity in clinical or procedural communication. In both modalities, surface plausibility may conceal errors that are difficult for a novice to recognize.

Terminological accuracy and anatomical accuracy are related but distinct. A correctly rendered structure may be given a non-standard or incorrect label; a TA2-preferred label may be attached to an anatomically incorrect structure; and an output may fail in both respects. Citation accuracy is a further issue because a plausible but fabricated source cannot validate either the terminology or the underlying anatomical claim.

Broader anatomy-specific governance has already been addressed in a recently published risk-tiered framework encompassing donor-derived material, anatomical fidelity, variation, assessment, provenance, human accountability, and lifecycle monitoring [4]. The present review does not propose a second general governance framework. It addresses a narrower research question: what evidence supports the TA2 compliance of GenAI outputs, where do verification gaps remain, and what level of human review is appropriate before those outputs are used?

The four-step checklist developed here is intended as a terminology-specific audit layer that can be applied within the broader framework [4]. It centers on term use, image labeling, source integrity, and multilingual standardization. It does not reproduce the earlier framework's architecture for donor-data authorization, assessment decisions, or lifecycle governance.

Accordingly, this review synthesizes evidence relevant to terminology compliance and proposes a proportionate verification checklist for educators, researchers, editors, and clinicians. It does not attempt to rank individual commercial models, whose versions and behavior change rapidly. Instead, it asks what currently available evidence supports, where important gaps remain, and what minimum human verification is appropriate before GenAI-generated anatomical material is used.

## Review

Methods

Search and Selection Approach

This structured narrative review was conducted and reported with reference to the Scale for the Assessment of Narrative Review Articles (SANRA) [5]. PubMed searches were initially performed on September 6-7, 2026 and rerun on September 19, 2026. Four domain-specific strategies addressed terminology and text, anatomical images, reference hallucination, and human or multilingual terminology. No lower publication-date limit was imposed; the upper limit was September 19, 2026. The exact final strategies and yields are reported in Table 1. Reference-list checking, targeted publisher searches, and official terminology and journal-policy websites supplemented PubMed.

**Table 1:** Reproducible PubMed search strategies and yields

Domain	Exact PubMed strategy rerun September 19, 2026	Yield
Terminology and text	("Terminologia Anatomica"[Title/Abstract] OR "anatomical terminology"[Title/Abstract] OR "anatomical nomenclature"[Title/Abstract]) AND ("generative artificial intelligence"[Title/Abstract] OR "large language model"[Title/Abstract] OR "large language models"[Title/Abstract] OR ChatGPT[Title/Abstract] OR Gemini[Title/Abstract]) AND ("1900/01/01"[dp] : "2026/09/19"[dp])	8
Anatomical images	(anatomy[Title/Abstract] OR anatomical[Title/Abstract]) AND ("text-to-image"[Title/Abstract] OR "image generation"[Title/Abstract] OR "AI-generated image"[Title/Abstract] OR "AI-generated images"[Title/Abstract]) AND (illustration[Title/Abstract] OR illustrations[Title/Abstract] OR "medical education"[Title/Abstract]) AND ("1900/01/01"[dp] : "2026/09/19"[dp])	23
References and hallucination	(anatomy[Title/Abstract] OR anatomical[Title/Abstract]) AND (ChatGPT[Title/Abstract] OR "large language model"[Title/Abstract] OR "large language models"[Title/Abstract]) AND (citation[Title/Abstract] OR citations[Title/Abstract] OR reference[Title/Abstract] OR references[Title/Abstract] OR hallucination[Title/Abstract]) AND ("1900/01/01"[dp] : "2026/09/19"[dp])	45
Human adoption and multilingual terminology	("Terminologia Anatomica"[Title/Abstract] OR "anatomical terminology"[Title/Abstract] OR "anatomical nomenclature"[Title/Abstract]) AND (usage[Title/Abstract] OR adoption[Title/Abstract] OR compliance[Title/Abstract] OR eponym*[Title/Abstract] OR multilingual[Title/Abstract] OR translation[Title/Abstract] OR language[Title/Abstract]) AND ("1900/01/01"[dp] : "2026/09/19"[dp])	128

Sources were eligible when they directly evaluated GenAI-generated text or images against TA/TA2 or anatomical correctness; quantified human or editorial adoption of standardized anatomical terminology; examined reference reliability, translation, or terminology learning relevant to anatomical governance; or supplied an authoritative terminology or publisher-policy statement. Disease and syndrome eponyms outside anatomical-structure nomenclature, image-to-image conversion studies unrelated to language translation, non-anatomical AI evaluations, and sources that did not permit verification of the relevant claim were excluded.

The four PubMed strategies returned 204 records in total and 198 unique records after deduplication. Because this was a structured narrative review rather than a systematic review, candidates were selected purposively for direct relevance and evidentiary value rather than processed as an exhaustive PRISMA flow. One human author made the final selection, classification, and interpretation decisions. During revision, bibliographic records and the key quantitative findings discussed in the text were rechecked against publisher or indexed records. No formal risk-of-bias instrument or duplicate screening process was applied.

Appraisal and synthesis were descriptive. Findings were grouped by evidence domain and interpreted according to study design, task, model version, outcome definition, and sample size. No meta-analysis or meta-regression was attempted because populations, prompts, model versions, anatomical regions, scoring rubrics, and outcomes were too heterogeneous for a meaningful pooled estimate. Unless otherwise specified, percentages below are study-specific point estimates rather than generalizable error rates. The previously published risk-tiered framework [4] supplied conceptual context but was not treated as primary evidence of TA2 compliance.

Results

Baseline Human and Editorial Compliance

Incomplete adoption of standardized terminology predates GenAI. North American surveys found that preferred standardized terms were the most frequently used option for only 44% of surveyed term sets in 2009 and 53% of surveyed structures or features in 2014; differences were also observed between professional associations [6-8].

Editorial and educational practice shows a similar gap. Among 16 responding anatomy-journal editors in 2024, 75% knew the TA2 recommendation concerning eponyms, but only 50% reported following it in their editorial office [9]. United Kingdom educator perspectives and recent commentary likewise document persistent conventional terms and terminological drift [10,11].

These findings should not be interpreted as a mathematical ceiling on GenAI performance. A model may sometimes normalize a synonym or correct a source inconsistency. Nevertheless, an inconsistent publication and teaching environment creates a plausible pathway by which outdated labels, regional preferences, and eponyms are reproduced. GenAI-related non-compliance should therefore be treated partly as a redistribution of pre-existing inconsistency and partly as a model-specific failure requiring direct verification.

Text-Based GenAI and Anatomical Terminology

Text studies differ substantially in task design and outcome definition. Some assess general anatomical knowledge through multiple-choice questions, whereas others explicitly evaluate terminology. Nahir et al. compared several chatbots using a 70-item examination translated in accordance with Terminologia Anatomica; the study supports model-dependent variability in anatomical knowledge but does not isolate TA2 terminology as a stand-alone endpoint [12].

More direct terminology measures have now appeared. Örs used a study-specific, author-developed Anatomical Terminology Accuracy (ATA) scale in a comparison of large language models answering clinical neuroanatomy cases [13]. No prior external validation of the ATA scale was identified; however, two raters applied the rubric and the study reported good inter-rater reliability for ATA (intraclass correlation coefficient = 0.857). Sivri et al. used Terminologia Anatomica version 2.07 as the reference standard when scoring radiological structure-identification responses from contemporary models [14]. These designs demonstrate that TA2 alignment can be measured explicitly rather than inferred from overall answer accuracy, while also showing why the measurement instrument itself must be scrutinized.

Performance remains task-dependent. In a practical examination using 265 structures marked on cadaveric photographs, ChatGPT-4o correctly identified 59 of 265 structures within three attempts (22.26%; 22.3% when rounded to one decimal place) and occasionally generated nonexistent anatomical terms [15]. The source used a combined correctness criterion: a response counted as correct only when it supplied a valid anatomical term that precisely corresponded to the marked structure. A separate gross-anatomy question study reported broad variation across systems [16]. Identifying a marked structure, answering an examination question, and selecting the preferred TA2 term are related but distinct tasks.

The defensible conclusion is therefore narrower than saying that a particular model "knows anatomy." Some systems can generate correct terminology in some contexts, and recent studies provide direct ways to score this. No reviewed study establishes reliable TA2 compliance across anatomical regions, prompt types, languages, and model versions. Model names and version numbers should always accompany reported results because performance cannot safely be generalized to earlier or later releases.

Image-Generation Fidelity and Labeling

Kozioł et al. evaluated 12,126 general and 2,503 detailed anatomical items generated using Midjourney and mapped them to TA2 identifiers. Structural presence was greater for detailed than general prompts (40.3% versus 21.7%; p < 0.001), physiological order was also higher (15.5% versus 4.6%; p < 0.001), and hallucinated items occurred in both conditions (4.9% versus 6.3%; p = 0.034) [17].

Zhang and Soares screened 4,734 manuscripts in a defined set of journals and identified 37 articles containing facial anatomical depictions. Four of the 37 articles (10.8%), all published in 2025, contained 12 AI-generated figures; all 12 of 12 were reported to display gross anatomical inaccuracies [18]. This estimate is bounded by the selected journal set, date range, and focus on facial anatomy.

Other direct evaluations reach a similar qualitative conclusion using different tools and criteria. Paşlı and Yücedağ Gündoğdu examined the potential and limitations of text-to-image generators for anatomical illustration [19]. Strugała et al. assessed 144 images from six generators against TA-based reference tables; DALL-E produced the highest combined proportion of structures both present and physiologically correct, but the proportion was 37.4% [20].

Additional studies have compared image generators across general anatomy, anatomy education, specific clinical presentations, neurosurgical contexts, and aesthetic-surgery applications [21-25]. They report recurring problems with topology, laterality, branching, proportions, omissions, inventions, and labeling. Their numerical results should not be compared directly because prompts, generators, regions, rubrics, and reviewer expertise differ.

Image verification must consequently address two layers. The first is terminology: whether each visible label is a current and appropriate TA2 term. The second is structural fidelity: whether the structure exists, is placed and related correctly, and is represented at an appropriate level of detail. Passing one layer cannot compensate for failure of the other.

Reference and Citation Integrity

Terminology claims are often accompanied by model-generated citations, but a citation-shaped string is not evidence until independently located. Ülkir and Paşlı submitted 120 anatomy questions across six categories to ChatGPT 5.2, Gemini 3 Pro, and DeepSeek V3.2 and examined 1,800 references. Hallucination rates were 23.2%, 45.8%, and 47.5%, respectively, while PubMed identifier accuracy ranged from 25.1% to 57.6%. Citation-content consistency differed significantly among models (p < 0.001) [26]. These labels describe the exact model versions evaluated and should not be generalized to later releases.

Transferable evidence outside anatomy reaches the same general warning. Chelli et al. found substantial hallucination and reference-accuracy problems when ChatGPT and Bard were used to generate content for systematic reviews [27]. These studies support a simple governance rule: an AI-generated citation should be treated as an unverified lead, not as a source. Authors should locate the publisher or indexed record, match the title and authors, confirm the DOI or identifier, and read the source before retaining the associated claim.

Multilingual Terminology

Direct evidence concerning TA2 compliance in non-English GenAI output remains sparse. Rath conducted an eight-week mixed-methods pilot involving 35 Chinese international dental students who used ChatGPT to support oral-anatomy terminology learning. Comprehension improved with a large reported within-group effect size (Cohen's d = 1.91), and students raised concerns about accuracy and overreliance [28]. The estimate should be interpreted cautiously because the study was small, uncontrolled, and vulnerable to practice, maturation, and regression-to-the-mean effects; it evaluated learning rather than direct TA2 compliance of model output.

A systematic review of AI-supported clinical translation found potential benefits alongside variable accuracy and consistency across tools and languages [29]. Earlier terminology work also shows that multilingual standardization is not a new challenge. Researchers have proposed multilingual extensions of Terminologia Anatomica, developed algorithms for adding French-language terms, audited nomenclature in Czech journals, and called for evidence-based standardization of Greek anatomical nomenclature [30-33]. These sources establish the importance of multilingual governance but do not quantify the accuracy of current GenAI systems.

Until direct comparative evidence becomes available, non-English anatomical terminology should be verified against an authoritative TA2-aligned bilingual resource or by a bilingual anatomist familiar with the target language. Literal translation from English is insufficient when established professional usage differs.

Table 2 separates what the evidence supports from what remains uncertain. This distinction matters because a practical safeguard may be reasonable even when it has not yet been validated as a formal instrument.

**Table 2:** Evidence landscape for GenAI anatomical terminology governance

Domain	What the evidence supports	Principal limitation
Human and editorial baseline	TA-preferred terminology is incompletely adopted, and editorial enforcement varies [6-11].	Surveys are geographically concentrated and use different samples.
Text outputs	TA2 alignment can be scored directly, and performance varies by model and task [12-16].	Few studies isolate terminology from general anatomical accuracy.
Image outputs	Anatomical omissions, inventions, relationship errors, and labeling problems recur across generators [17-25].	Prompts, regions, rubrics, and model versions are heterogeneous.
References	Anatomy-specific and transferable studies document substantial citation fabrication and metadata errors [26,27].	Only one reviewed study is anatomy-specific.
Multilingual output	Multilingual learning may benefit from AI support, but terminology accuracy remains uncertain [28-33].	Direct non-English TA2-output studies are lacking.
Publication policy	Substantive AI use requires disclosure and human accountability; image rules may be restrictive [34-38].	Publisher policies change and do not themselves verify terminology.

Editorial and Policy Considerations

Publication policies address authorship, disclosure, accountability, and image provenance more directly than terminology accuracy. Table 3 summarizes the current positions of the bodies and publishers reviewed here [34-38].

**Table 3:** Comparison of current AI publication policies

Publisher or body	AI authorship	Disclosure	AI-generated images
Cureus [34]	Not permitted	Substantive use must be documented	Generally prohibited, with limited case-by-case exceptions
ICMJE [35]	Not permitted	Tool and purpose should be disclosed	Journal policy applies; humans remain accountable for accuracy
COPE [36]	Not permitted	Use in writing, images, or data work should be disclosed	Human authors retain responsibility and liability
Elsevier [37]	Not permitted	Separate declaration; image-specific disclosure where applicable	Certain explanatory images allowed; primary research images cannot be created or altered; graphical abstracts restricted
Springer Nature [38]	Not permitted	Permitted use must be labeled	Generally prohibited, with narrow exceptions

The policies converge on human accountability, exclusion of AI tools from authorship, and disclosure of substantive use, but differ in their treatment of generated images. Therefore, disclosure, permission to publish an image, and verification of TA2 terminology remain separate controls. Authors must check the policy of the specific journal close to submission because these requirements change rapidly.

A Proposed Verification Checklist

Four-step checklist:** **The checklist translates the terminology-specific evidence into a minimum review process and complements the broader risk-tiered framework [4]. Risk classification determines verification intensity, while this checklist specifies what must be checked at the level of terms, structures, and sources. It is an operational aid rather than a validated measurement scale. The four steps and minimum completion criteria are summarized in Table 4.

**Table 4:** Four-step verification checklist for GenAI-generated anatomical content

Step	Required action	Minimum completion criterion
1. Classify the output and its risk	Record whether the material is text, labels, references, assessment content, or an image; identify the audience and intended use.	The use is assigned a low-, moderate-, or high-consequence category before release.
2. Verify terminology	Check every anatomical name against TA2 or an authoritative TA2-aligned resource; distinguish preferred terms, accepted synonyms, eponyms, and obsolete forms.	Every retained term is confirmed or deliberately explained; uncertain terms are removed.
3. Verify anatomy and sources	For text, confirm factual claims and every cited source. For images, separately check presence, position, laterality, relationships, branching, proportions, labels, and clinically relevant variants.	A qualified human reviewer can trace each substantive claim or structure to an authoritative source.
4. Document accountability	Record the tool and version when available, the role of AI, the human reviewer, major corrections, and the applicable disclosure or image policy.	The responsible human approves the final output, and the required disclosure is included.

Proportionate Application

The amount of verification should follow the consequence of error. Low-consequence uses include private brainstorming, early outlines, or non-distributed drafts; a knowledgeable user should check such material before it informs later work, although a formal audit trail is usually unnecessary. Moderate-consequence uses include student handouts, slides, public education, formative questions, or labeled diagrams, and these warrant a higher standard: every label, factual claim, and reference should be checked by a subject-matter expert, and the tool's role should be documented according to institutional or publisher policy. High-consequence uses include summative assessment, peer-reviewed publication, clinical or procedural guidance, surgical planning, or donor-derived material; for these, verification should be complete and traceable, preferably with a second qualified reviewer for images or other high-stakes content, and material that cannot be independently verified should not be used.

This approach is deliberately output-centered. A familiar model name, polished appearance, or successful performance on an unrelated benchmark does not reduce the required review. Conversely, the checklist does not prohibit low-risk exploratory use when the output remains clearly provisional.

Discussion

Practical Implications

The consequence tiers above provide the underlying logic for role-specific application. Authors should verify every retained source during drafting by matching its bibliographic record and reading the passage that supports the claim. Citation managers can format a verified record but cannot establish that it supports the sentence.

Educators should not distribute unreviewed AI-generated diagrams or terminology lists. Comparing GenAI output with TA2 can be a useful critical-appraisal exercise when an expert-confirmed answer is available. Moderate-consequence teaching material requires complete checking of labels, claims, and references; high-consequence summative assessment requires traceable review because ambiguous terminology may change the intended correct answer.

Editors and reviewers should evaluate terminology, structural fidelity, source traceability, and disclosure separately. Attractive presentation or a correct caption cannot substitute for structure-by-structure image review, and correct general meaning cannot excuse an obsolete label, misleading eponym, or fabricated reference. Disclosure identifies AI involvement but does not itself provide quality assurance.

Limitations

This structured narrative review remains subject to selection bias. Its search was centered on PubMed and supplemented by citation checking and targeted official-source searches; subscription databases and a formal grey-literature search were not used. Exact PubMed strategies and yields are now reported, but selection was purposive rather than a systematic, duplicate-screened process. Screening and classification were completed by one human author, without inter-rater reliability testing. No formal risk-of-bias instrument was applied.

Some included studies appeared online in 2025-2026 and may not yet have stable issue or page assignments. Model-specific results can become outdated quickly. Several text studies assessed general anatomical performance rather than terminology as an independent endpoint, and image studies used varied prompts and scoring frameworks. Evidence for multilingual TA2 output and the real-world effect of verification procedures remains particularly limited.

Finally, the four-step checklist is author-proposed. Its content is grounded in recurrent failure modes and existing accountability requirements, but its reliability, usability, and effect on error reduction have not been prospectively validated. Future studies should test it across users with different levels of anatomical expertise and across text, image, multilingual, assessment, and publication tasks.

The review is conceptually adjacent to a previously published anatomy-governance framework [4]. The present unit of analysis is narrower, but the added value and usability of embedding this terminology-specific checklist within the broader framework have not yet been evaluated empirically.

## Conclusions

Available evidence does not establish that general-purpose GenAI produces reliably TA2-compliant anatomical content. Text models can answer correctly while using inconsistent terminology, image generators can combine visual realism with structural or labeling errors, and generated references can be fabricated or bibliographically inaccurate. These risks arise within a human-authored literature that already shows incomplete adoption of standardized terminology.

A proportionate response is therefore preferable to either uncritical adoption or blanket rejection. Users should classify the intended use, verify every anatomical term, independently check scientific content and sources, and document accountable human review and AI disclosure. The verification burden should rise with the consequence of error. For assessment, publication, and clinical or procedural use, unverified GenAI-generated anatomical content should not be accepted. This terminology-specific audit should complement, rather than replace, broader controls for donor data, assessment, accountability, and lifecycle monitoring.
